# Analysis of Phosphatase Activity in a Droplet-Based Microfluidic Chip

**DOI:** 10.3390/bios12090740

**Published:** 2022-09-08

**Authors:** Bala Murali Krishna Vasamsetti, Yeon-Jun Kim, Jung Hoon Kang, Jae-Won Choi

**Affiliations:** 1 Department of Biomedical Science, Cheongju University, Cheongju 28160, Korea; 2 Toxicity and Risk Assessment Division, Department of Agro-Food Safety and Crop Protection, National Institute of Agricultural Sciences, Rural Development Administration, Wanju-gun 55365, Korea; 3 Department of Bioindustrial Engineering, Cheongju University, Cheongju 28503, Korea

**Keywords:** droplet-based microfluidics, microdroplet, phosphatase activity, PTP assay, DUSP22, ethyl-3,4-dephostatin

## Abstract

We report analysis of phosphatase activity and inhibition on droplet-based microfluidic chips. Phosphatases are such attractive potential drug targets because abnormal phosphatase activity has been implicated in a variety of diseases including cancer, neurological disorders, diabetes, osteoporosis, and obesity. So far, several methods for assessing phosphatase activity have been reported. However, they require a large sample volume and additional chemical modifications such as fluorescent dye conjugation and nanomaterial conjugation, and are not cost-effective. In this study, we used an artificial phosphatase substrate 3-*O*-methylfluorescein phosphate as a fluorescent reporter and dual specificity phosphatase 22. Using these materials, the phosphatase assay was performed from approximately 340.4 picoliter (pL) droplets generated at a frequency of ~40 hertz (Hz) in a droplet-based microfluidic chip. To evaluate the suitability of droplet-based platform for screening phosphatase inhibitors, a dose–response inhibition study was performed with ethyl-3,4-dephostatin and the half-maximal inhibitory concentration (IC_50_) was calculated as 5.79 ± 1.09 μM. The droplet-based results were compared to microplate-based experiments, which showed agreement. The droplet-based phosphatase assay proposed here is simple, reproducible, and generates enormous data sets within the limited sample and reagent volumes.

## 1. Introduction

Protein phosphorylation, a post-translational modification, is an important process in human health and disease controlled by the coordinated activity of enzymes known as kinases and phosphatases, which add and remove phosphate groups from proteins, respectively [1]. The regulation of physiological processes including gene expression, cell proliferation and differentiation, cell cycle arrest, and apoptosis largely depends on the phosphorylation of serine, threonine, and tyrosine residues in eukaryotic proteins [2].

The dual specificity phosphatases (DUSPs) are cysteine-based phosphatases of the protein tyrosine phosphatase (PTP) superfamily. As essential regulators of intracellular signaling events, DUSPs have been implicated in multiple biological processes and diseases [3]. DUSPs dephosphorylate both threonine/serine and tyrosine residues of their substrates, and some of them also play a role in MAPK signaling pathways [4]. Mitogen-activated protein kinases (MAPKs) are important participants in signal transduction pathways, and the control of these MAPK family members determines the fate of cellular homeostasis [5]. Several PTPs have emerged as key human oncogenes and are now being evaluated as potential therapeutic targets in addition to maintaining cellular homeostasis [6,7]. Alteration in the expression of CDC25C, a phosphatase, leads to genomic instability and often promotes more aggressive tumor development [8]. PTP1B, a tyrosine phosphatase, is a negative regulator of the insulin and leptin receptor pathways [9,10]. SHP-1 protein tyrosine phosphatase expression has been shown to be elevated in breast, ovarian, and prostate cancer, and Rodriguez et al. showed that SHP-1 knockdown inhibits G1/S progression in prostate cancer [11]. In thyroid tumors with BRAF mutations, DUSPs have been described as markers of higher MAPK signaling activation [12]. Since phosphatases are recognized as molecular markers for various cancers and as a viable target for cancer therapy, a high-throughput assay for detecting phosphatase activity and screening for phosphatase inhibitors is needed.

Phosphatase activity is typically measured using colorimetric methods that detect free phosphate and are based on a classic malachite green. So far, various phosphatase assays have been performed using malachite green [13], *p*-nitrophenyl phosphate [14], inositol 1,3,4,5-tetrakisphosphate, phosphatidylinositol-3,4,5-trisphosphate [15], and 3-*O*-methylfluorescein phosphate [16,17]. A label-free protein tyrosine phosphatase assay using a peptide substrate has also been described [18]. To this day, these traditional methods have made a significant contribution to solving the mystery of the various phosphatases. Nevertheless, these methods have limitations when applied to high-throughput analysis, as they require large sample volumes (hundreds of microliters to milliliters) and more time for analysis.

Droplet-based microfluidic techniques have become increasingly popular in recent years as they offer several advantages over traditional techniques, including the ability to automate handling procedures and miniaturize sample sizes [19,20]. In droplet-based microfluidics, an aqueous and an oil phase are rapidly mixed in a microfluidic device to produce water-in-oil emulsion droplets at kilohertz (kHz) frequencies. Lab-on-a-chip technologies have been successfully demonstrated in the fields of pharmacology, cell biology, and biochemistry [21]. Using droplet-based microfluidic system, we have previously reported protein–protein interactions analysis [22], SNP detection [23], RNase activity assay [24], and DNase activity assay [25]. Therefore, to identify phosphatases and screen for phosphatase inhibitors, droplet-based microfluidics has the potential to be a low-cost, high-throughput screening technology.

To address the disadvantages of the phosphatase assays described above, in the present study, the 3-*O*-methylfluorescein phosphate (OMFP)-based phosphatase assay was optimized and miniaturized in a droplet-based microfluidic platform for adaptation to high-throughput analysis of phosphatase and phosphatase inhibitors. As a model system, the enzymatic activity of DUSP22 and its inhibition by ethyl-3,4-dephostatin was measured in sub-nanoliter droplets. The method proposed here is simple, reproducible, and inexpensive.

## 2. Materials and Methods

### 2.1. Materials

Ethyl-3,4-dephostatin and 3-OMFP were purchased from Sigma-Aldrich (St. Louis, MO, USA) and dissolved in DMSO and sterile water, respectively. Polydimethylsiloxane (PDMS, Sylgard 184 silicone elastomer kit) was purchased from Dow Corning (Midland, MI, USA), mineral oil was purchased from Sigma-Aldrich, and ABIL EM 90 surfactant was purchased from Evonik Industries (Essen, Germany). A disposable 1.0 mm diameter biopsy punch was purchased from Integra Lifesciences (Princeton, NJ, USA). Glass slides (76 × 26 mm) with a thickness of 1 mm were obtained from Paul Marienfeld (Lauda-Königshofen, Germany). Fluorometric measurements in a microplate were performed using a SpectraMax M5e multi-mode microplate reader from Molecular Devices (San Jose, CA, USA).

### 2.2. Purification of DUSP22 Protein

The BL21(DE3) strain *E. coli* cells containing pET-28a(+)-DUSP22 were cultured in LB broth and induced with 0.5 mM isopropyl β-D-1-thiogalactopyranoside at 18 °C overnight. After induction, bacterial cells were centrifuged at 13,000 rpm/4 °C. The bacterial pellet was dissolved in the appropriate volume of lysis buffer (20 mM Tris-HCl (pH 8.0), 0.5 M NaCl and 5 mM imidazole, and 1 mM PMSF), and then lysed by sonication. The lysate was centrifuged at 5000 rpm for 30 min at 4 °C and the supernatant was transferred to the gravity flow column packed with Ni-NTA His-Bind^®^ Resin from Merck Millipore (Burlington, MA, USA). The column was washed (20 mM Tris-HCl (pH 8.0), 0.5 M NaCl, 50 mM imidazole) and eluted (20 mM Tris-HCl (pH 8.0), 0.5 M NaCl, 300 mM imidazole). The isolated protein buffer was substituted with 30 mM Tris-HCl (pH 8.0) in Amicon Ultra–0.5 Centrifugal Filter Unit (MWCO: 10 kDa) from Merck Millipore. The extracted DUSP22 was stored at −70 °C with 30% glycerol until use.

### 2.3. Fabrication of Microfluidic Devices and Operation

The microfluidic device was designed and prepared based on our previous reports [24,25]. A simple T-junction PDMS microfluidic device featuring an oil inlet, a sample inlet, and an outlet was fabricated using standard soft lithography techniques. The required amounts of PDMS prepolymer and curing agent (10:1, weight ratio) were thoroughly mixed and cast onto a microchannel patterned Si wafer mold. A semi-cured PDMS block was punched with two inlets and an outlet hole with a 1.0 mm diameter micro-punch. The glass slides and punched PDMS blocks were cleaned by sonication, treated with oxygen plasma for 40 s, and the PDMS blocks immediately bonded to the glass slides. The bonded microfluidic devices were kept on a 70 °C hot plate for 1 h prior to use in the experiments.

An aqueous inlet was used to inject the samples for analysis of DUSP22 activity. Mineral oil with 0.2% (*w*/*w*) ABIL EM 90 surfactant was used as the carrier liquid. A precision syringe pump from Harvard Apparatus (PHD2000, Holliston, MA, USA) was used to operate the microfluidic device at a constant sample flow rate of 1.0 µL/min and oil flow rate of 1.0 µL/min. Emitted fluorescence signal from microdroplets was detected using an electron multiplying-charge coupled device (EM-CCD) from Princeton Instruments (Trenton, NJ, USA) and an inverted fluorescence microscope from Olympus (IX71, Shinjuku, Japan) equipped with a 488 nm diode laser (10 mW) from World Star Tech (Markham, ON, Canada) as a light source. The fluorescence intensity of each microdroplet was collected using WinSpec/32 software from Princeton Instruments. Droplet generation frequency and fluorescence intensity results were evaluated using the WinSpec/32 software.

### 2.4. Droplet-Based Microfluidic Fluorescence Measurements

A microfluidic device with a simple T-junction geometry was used to generate droplets. The width and height of the microchannels in the droplet-based microfluidic chip were 50 µm and 50 µm, respectively. The carrier fluid was mineral oil containing 0.2% (*w*/*w*) ABIL EM 90 surfactant. The activity and inhibition of DUSP22 were monitored in 30 mM Tris-HCl (pH 7.0) buffer containing 1 mM EDTA, 0.1 mM DTT, 75 mM NaCl, and 0.33% BSA. For the phosphatase assay, the different concentrations (0, 0.98, 1.95, 3.91, 7.81, 15.63, 31.25, 62.50, 125, and 250 nM) of DUSP22 were mixed in the buffer. The reaction was started by the addition of 3-OMFP to a final concentration of 10 µM. Droplets were generated, and fluorescence intensity was detected 20 min after the addition of 3-OMFP. For the phosphatase inhibition assay, various concentrations (0.39, 0.78, 1.56, 3.13, 6.25, and 12.5 µM) of ethyl-3,4-dephostatin are mixed in buffer containing DUSP22 of 100 nM. The reaction is started by the addition of 3-OMFP to a final concentration of 10 µM. Droplets were generated, and fluorescence intensity was detected after 20 min incubation. Fluorescence intensity data was extracted using the WinSpec/32 software.

### 2.5. Microplate-Based Fluorescence Measurements

Microplate-based phosphatase activity and inhibition assays were conducted in a 96-well microplate using 30 mM Tris-HCl (pH 7.0) buffer containing 1 mM EDTA, 0.1 mM DTT, 75 mM NaCl, and 0.33% BSA under dark conditions. All assays were performed in a final reaction volume of 200 µL with the addition of 3-OMFP initiating the reactions. The phosphatase activity assay was performed by measuring the dephosphorylation of the 3-OMFP substrate. The reaction was initiated by the addition of 3-OMFP to a final concentration of 10 µM to a buffer containing various concentrations of DUSP22 (0, 5, 10, 20, 40, 60, 80, 100, 150, and 200 nM). Samples were incubated at room temperature and fluorescence emission was measured after 20 min using a SpectraMax M5e microplate reader with an excitation filter and an emission filter set at 488 nm and 515 nm, respectively. The phosphatase inhibition assay was performed by adding 3-OMFP (final concentration of 10 µM) to a buffer containing various concentrations of ethyl-3,4-dephostatin (0.39, 0.78, 1.56, 3.13, 6.25, and 12.5 µM) and 100 nM DUSP22, and the fluorescence intensity was measured after 20 min under the same excitation and emission conditions as in the phosphatase activity assay.

## 3. Results and Discussion

### 3.1. Working Principle of Phosphatase Activity Analysis in a Droplet-Based Microfluidic Chip

**Figure 1:**
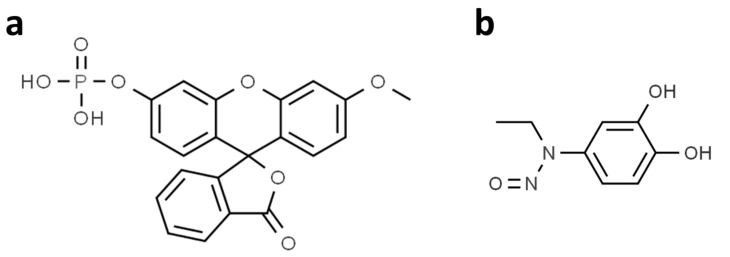
Structure of phosphatase substrate and phosphatase inhibitor used to analyze DUSP22 activity. (**a**) Structure of 3-OMFP, a substrate for DUSP22. (**b**) Structure of ethyl-3,4-dephostatin, an inhibitor of DUSP22 activity.

Droplet-based phosphatase activity assay (dPAA) and droplet-based phosphatase inhibition assay (dPIA) were demonstrated in picoliter droplets using 3-OMFP as a substrate (Figure 1a), DUSP22 as an enzyme, and ethyl-3,4-dephostatin as a phosphatase inhibitor (Figure 1b). 3-OMFP, a fluorescein derivative, has proven to be a useful tool for several enzymatic studies due to its high fluorescence properties and structural similarity to ATP [16]. Ethyl-3,4-dephostatin, a stable synthetic analog of dephostatin that is a potent inhibitor of intracellular PTPs and acts as a multi-phosphatase inhibitor by binding to the phosphatase catalytic site [26]. DUSP22 proteins for phosphatase assay were purified by immobilized metal ion affinity chromatography from *E. coli* carrying the pET-28a(+)-DUSP22 plasmid (Figure S1, Supplementary Materials).

**Figure 2:**
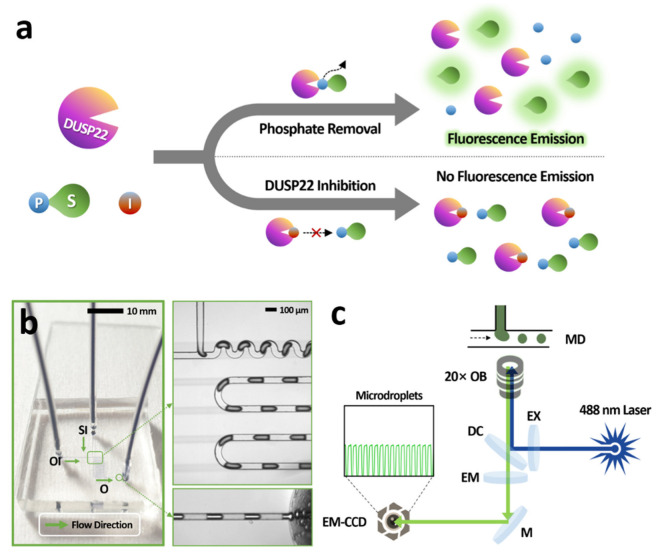
(**a**) Schematic representation of the working principle of phosphatase activity and inhibition assays (S, substrate; I, inhibitor; P, phosphate). (**b**) A droplet-based microfluidic chip with a simple T-junction structure was used for the analysis of DUSP22 activity. SI, sample inlet; OI, oil inlet; and O, outlet; Arrowhead, flow direction of liquid. (**c**) Optical setup for analyzing the fluorescence signal from microdroplets in a microfluidic channel (MD, microfluidic device; OB, objective; EX, excitation filter; DC, dichroic mirror; EM, emission filter; M, mirror; EM-CCD, electron multiplying-charge coupled device).

The proposed phosphatase assay is based on the detection of fluorescence emission from 3-*O*-methylfluorescein (3-OMF) at 515 nm with an excitation wavelength set at 488 nm. The enzyme DUSP22 dephosphorylates the 3-OMFP, resulting in the release of a highly fluorescent 3-OMF (Figure 2a). When ethyl-3,4-dephostatin is present in the reaction, it binds to the DUSP22 catalytic site and inhibits the dephosphorylation of 3-OMFP by DUSP22, thereby reducing fluorescence.

The microfluidic chip used for the phosphatase assay is shown in Figure 2b. Polydimethylsiloxane (PDMS) chip with an aqueous inlet, an oil inlet, and single outlet was used to perform droplet-based microfluidic measurements. Samples to be analyzed were injected through the aqueous inlet, and carrier fluid was injected through the oil inlet. Due to its simple T-junction geometry, the microfluidic device enabled rapid droplet generation and detection. Droplets were created at ~40 Hz frequencies with 0.5 as the water fraction (*W_f_*) value and a total flow rate of 2 µL/min in all experiments. *W_f_* value can be used to adjust the size and volume of the droplets,

(1) $$W_{f}=V_{W}/\left(V_{W}+V_{O}\right),$$

where *V_w_* refers to a flow rate (μL/min) of the water phase and *V_o_* is a flow rate (μL/min) of the immiscible oil phase [27]. As shown in Figure 2b, the microdroplets generated inside the microchannel are monodispersed, and their volumes were calculated from the cuboid and sphere occupying the microchannel.

A schematic optical setup used for fluorescence intensity measurements is shown in Figure 2c. A 488 nm laser is used as the excitation source and the fluorescence emission from droplets was recorded with an EM-CCD detector.

### 3.2. Droplet-Based Phosphatase Activity Assay (dPAA)

Prior to droplet-based analysis of DUSP22 activity, dephosphorylation of 3-OMFP by DUSP22 was monitored in a microwell plate by a fluorescence spectrophotometer. Different concentrations of DUSP22 were added to a fixed amount of 3-OMFP (10 µM) and the rate of dephosphorylation was measured. The results showed a linear increase in the rate of dephosphorylation of 3-OMFP with increasing DUSP22 concentration, indicating that the assay is quantitative (Figure S2).

**Figure 3:**
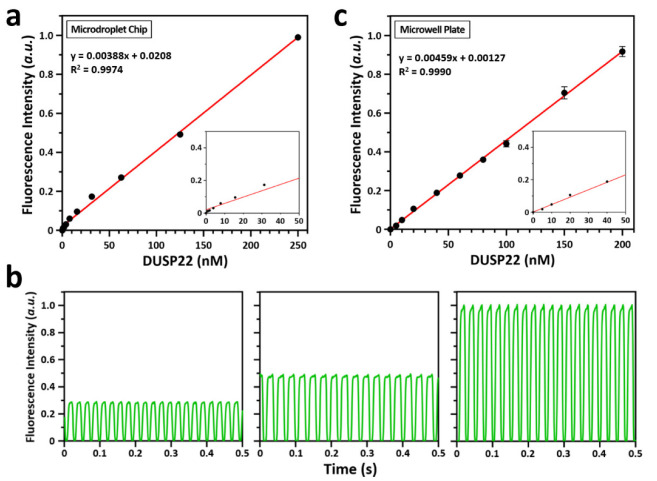
Fluorescence intensity analysis resulted from 3-OMFP degradation at designated concentrations of DUSP22. (**a**) Analysis of DUSP22 activity in a microdroplet chip. The concentration of 3-OMFP was fixed at 10 μM, and the concentration of DUSP22 was varied (0, 0.98, 1.95, 3.91, 7.81, 15.63, 31.25, 62.50, 125, and 250 nM). The error bars are smaller than the black circle of each concentration. Inset shows fluorescence intensity for DUSP22 below 50 nM. (**b**) Examples of fluorescence intensity scanned for 0.5 s from ~340.4 pL droplets generated within a microdroplet chip. From the left, the concentrations of DUSP22 are 62.5, 125, and 250 nM, respectively. (**c**) Analysis of DUSP22 activity in a microwell plate. The concentration of 3-OMFP was fixed at 10 μM, and the concentration of DUSP22 was varied (0, 5, 10, 20, 40, 60, 80, 100, 150, and 200 nM). Some error bars not shown are smaller than the black circle of each concentration. Inset shows fluorescence intensity for DUSP22 below 50 nM.

To demonstrate the dPAA, various concentrations of DUSP22 (0 to 250 nM) were added to a fixed concentration of 3-OMFP substrate (10 µM). After 20 min incubation at room temperature, the samples were injected onto a microfluidic chip, and droplets were generated in a steady state. To generate monodisperse droplets at the T-junction (Figure 2b), both the sample and the oil were injected at a constant flow rate of 1.0 μL/min using a precision syringe pump. Fluorescence intensity emitted from the droplets was measured using an EM-CCD. The results showed that the fluorescence intensity increased with increasing concentration of DUSP22, suggesting that the droplet-based detection system can detect and quantify activity of phosphatases (Figure 3a). It showed excellent linearity at different concentrations of DUSP22. The linear equation for linearity was y = 0.00388x + 0.0208 and the R^2^ value was 0.9974. The dynamic linear range was between 1.95 nM and 250 nM of DUSP22, and the limit of detection (LOD) for DUSP22 was 1.95 nM. Figure 3b shows examples of the fluorescence peaks scanned for 0.5 s from droplets at different DUSP22 concentrations. The standard deviation of the results obtained using dPAA was negligible, so the data points in the graph showed almost no error bars. Taken together, these results indicate that the droplets formed at same time intervals and resulted in uniform fluorescence intensity.

Simultaneously, we also performed a microwell plate-based phosphatase assay (mPAA) under similar experimental conditions and the obtained results were compared with the dPAA results. Results from mPAA showed better agreement with dPAA (Figure 3c). Like the dPAA, it also showed excellent linearity at different concentrations of DUSP22. The linear equation for linearity was y = 0.00459x + 0.00127 and the R^2^ value was 0.9990. The dynamic linear range was between 5.0 nM and 200 nM of DUSP22, and the LOD for DUSP22 was 5.0 nM.

One of the main advantages of droplet-based experiments is the relatively small analytical sample volume requirement compared to conventional methods [28]. Here, the mPAA consumed 600 µL of analytical sample to obtain a valid reading (in triplicates) at a given concentration of DUSP22. On the other hand, the dPAA consumed about 14 nL of analytical sample to obtain a valid reading (40 replicates), which is about 43,000 times less than mPAA. For fluorescence measurements, our dPAA read about 40 samples per second, while the mPAA read 1 sample per 1.32 s. Overall, our results showed that dPAA outperformed mPAA in terms of generating multiple reads (monodispersed droplets) with a small sample size, obtaining faster, more accurate results.

### 3.3. Droplet-Based Phosphatase Inhibition Assay (dPIA)

**Figure 4:**
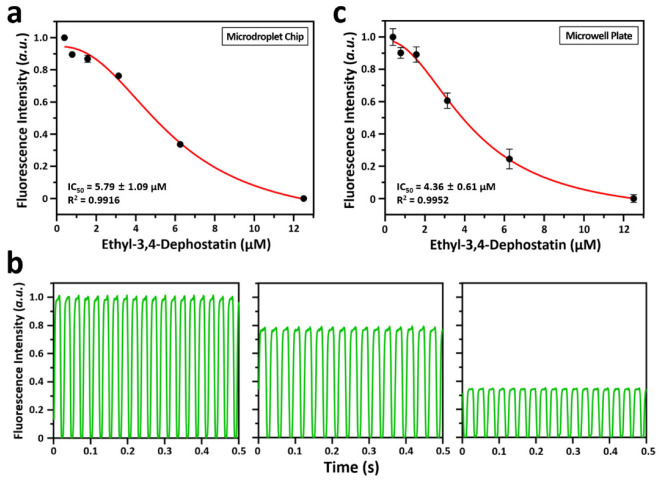
Fluorescence intensity analysis at a fixed concentration of 3-OMFP, DUSP22, and at designated concentrations of ethyl-3,4-dephostatin. (**a**) Analysis of inhibition of DUSP22 activity in a microdroplet chip. The concentrations of DUSP22 and 3-OMFP were fixed at 100 nM and 10 μM, respectively, and the concentration of ethyl-3,4-dephostatin was varied (0.39, 0.78, 1.56, 3.13, 6.25, and 12.5 µM). Some error bars not shown are smaller than the black circle of each concentration. (**b**) Examples of fluorescence intensity scanned for 0.5 s from ~340.4 pL droplets generated within a microdroplet chip. From the left, the concentrations of ethyl-3,4-dephostatin are 0.39, 3.13, and 6.25 μM, respectively. (**c**) Analysis of inhibition of DUSP22 activity in a microwell plate. The concentrations of DUSP22 and 3-OMFP were fixed at 100 nM and 10 μM, respectively, and the concentration of ethyl-3,4-dephostatin was varied (0.39, 0.78, 1.56, 3.13, 6.25, and 12.5 µM).

The enzyme inhibition assay is essential in high-throughput drug screening since several samples must be screened. Droplet-based microfluidic system can be used as a platform to perform enzyme inhibition assays due to their advantages of rapid mixing of reagents and low reagent/sample consumption [24,25]. Here, dPIA was demonstrated through a simple dose–response assay using ethyl-3,4-dephostatin as the phosphatase inhibitor (Figure 4a). The fluorescence peaks of representative droplets at different phosphatase inhibitor concentrations are shown in Figure 4b. The dPIA results confirmed that fluorescence intensity values decreased with increasing concentration of ethyl-3,4-dephostatin. This is because ethyl-3,4-dephostatin inhibits DUSP22 and thereby prevents the dephosphorylation of the 3-OMFP substrate. In addition, using the information from each inhibitor concentration, Equation (2) was used to determine the half-maximal inhibitory concentration (IC_50_) of that inhibitor, which was obtained as 5.79 ± 1.09 μM.

(2) $$Fluorescence\;Intensity=\{\left(I_{MAX}-I_{min}\right)/\left(1+e^{\left({\mathrm{IC}}_{50}-x_{0}\right)/dx}\right)\}+I_{min}$$

where *I_MAX_* means the fluorescence intensity of maximal in experiment, *I_min_* means the fluorescence intensity of minimal in experiment, and *x*_0_ means the concentration of ethyl-3,4-dephostatin when it has *I_min_*. In the fitted curve of dPIA, R^2^ value was 0.9916. Further, to validate dPIA, the results from dPIA were compared to conventional microwell plate-based phosphatase inhibition assay (Figure 4c). The inhibitory effect of ethyl-3,4-dephostatin against DUSP22 was measured in a microwell plate under the same reagent conditions as for dPIA and similar results were observed. In the fitted curve from microwell plate measurements, IC_50_ and R^2^ values were obtained as 4.36 ± 0.61 μM and 0.9952, respectively. While microwell plates consumed 600 µL of analytical sample to obtain a valid reading (triplicates) at a given ethyl-3,4-dephostatin concentration, the dPIA consumed approximately 14 nL of analytical sample to obtain a valid reading (40 replicates). Therefore, compared to the multiwell plate approach, the dPIA proposed here is superior in terms of generating large amounts of data with fewer samples and thus more cost-effective.

## 4. Conclusions

We demonstrated a simple assay for detecting phosphatase activity and screening phosphatase inhibitors using droplet-based microfluidics. Our results showed that droplet-based and microplate-based fluorescence measurements were comparable, suggesting that phosphatase detection and phosphatase inhibitor screening can be performed using fluorescence detection in conjunction with droplet-based microfluidics. We have successfully monitored the activity of DUSP22 using our droplet-based microfluidic device with 340.4 pL of analytical samples per measurement, which is about 587,000 times less than the 200 µL required for microwell plate-based experiments. While microwell plate-based methods read a sample every 1.32 s, our droplet-based phosphatase assay reads approximately 40 samples per second. For dPAA and dPIA, thousands of picoliter-sized droplets were generated and detected in a microfluidic chip, minimizing the use of expensive materials such as enzymes, substrates, and inhibitors. The high-throughput analytical method proposed here is simple, reproducible, and cost-effective. Despite these advantages, this study has a limitation. Although we used DUSP22 as a model to analyze phosphatase activity, the point is that many phosphatases, including DUSP22, exist in cancer cells [6,7,8,11,12,29,30]. Therefore, the separation of certain phosphatases is required for the real samples, such as cancer cells. Finally, we believe that our proposed platform will be an excellent tool for discovering various factors related to real-time phosphatase kinetics when combined with droplet-based microfluidics designed for long-term incubation from tens of minutes to several hours.

## Data Availability

All data used for the within the manuscript.

## Supplementary Materials

The following supporting information can be downloaded at: https://www.mdpi.com/article/10.3390/bios12090740/s1, Figure S1: Polyacrylamide gel image confirming recombinant DUSP22 proteins; Figure S2: Fluorescence intensities at designated concentrations of DUSP22 in a microwell plate.
