# Supplementary material for: Analysis of Phosphatase Activity in a Droplet-Based Microfluidic Chip
Source: Biosensors (Basel). 2022 Sep 8;12(9):740. doi: 10.3390/bios12090740 (PMC9496282; doi:10.3390/bios12090740)
Supplement: Supplementary file 1 [file biosensors-12-00740-s001.zip › biosensors-1861966-supplementary.pdf]

Supplementary Materials

Analysis of Phosphatase Activity in a Droplet-Based Microfluidic Chip

Bala Murali Krishna Vasamsetti [1,2], Yeon-Jun Kim [1], Jung Hoon Kang [1] and Jae-Won Choi [1,3,*]

1 Department of Biomedical Science, Cheongju University, Cheongju 28160, Republic of Korea
2 Toxicity and Risk Assessment Division, Department of Agro-Food Safety and Crop Protection, National Institute of Agricultural Sciences, Rural Development Administration, Wanju-gun 55365, Republic of Korea
3 Department of Bioindustrial Engineering, Cheongju University, Cheongju 28503, Republic of Korea
* Correspondence: jwchoi0211@gmail.com; Tel.: +82-43-229-8528

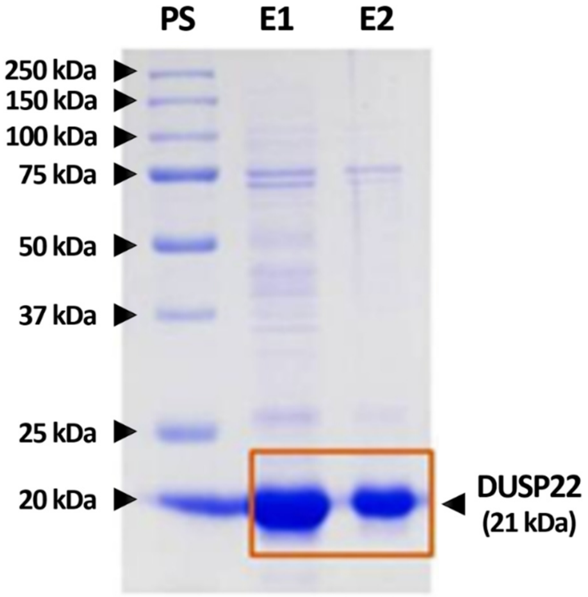

Figure S1. Polyacrylamide gel image confirming recombinant DUSP22 proteins. The recombinant DUSP22 proteins (6X His-tagged form) from a bacterial lysate were purified utilizing immobilized metal chromatography (IMAC) using Ni-NTA resins. Five microliter samples from the first two Ni-NTA resin packed column eluents were run on a 12% polyacrylamide gel and stained with Coomassie Brilliant Blue R-250. Lane 1: All Blue Prestained Protein Standard (PS) from Bio-Rad, Lane 2: DUSP22 elute 1 (E1), and Lane 3: DUSP22 elute 2 (E2).

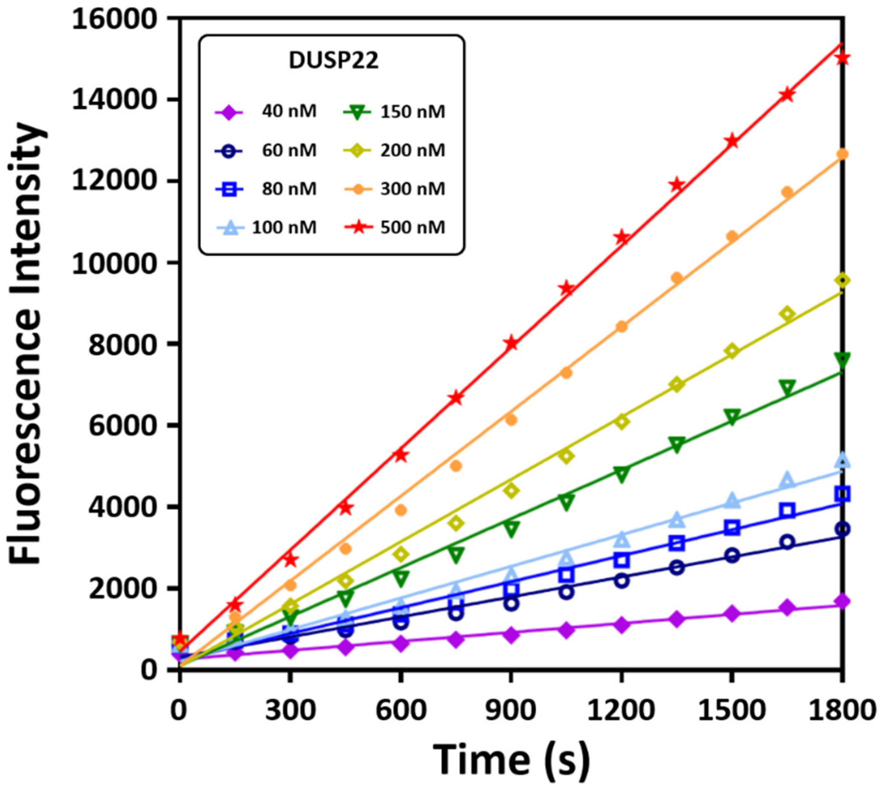

Figure S2. Fluorescence intensities at designated concentrations of DUSP22 in a microwell plate. The concentrations of DUSP22 were varied from 40 nM to 500 nM and the concentration of 3-OMFP was fixed at 10 μM. All samples were excited at 488 nm and fluorescence emission from the samples was monitored at 515 nm in a black 96-well plate.
